# Draft Genome Sequences of Eight Obligate Methane Oxidizers Occupying Distinct Niches Based on Their Nitrogen Metabolism

**DOI:** 10.1128/genomeA.00421-16

**Published:** 2016-08-04

**Authors:** Kim Heylen, Paul De Vos, Bram Vekeman

**Affiliations:** Department of Biochemistry and Microbiology, Laboratory of Microbiology, Ghent University, Ghent, Belgium

## Abstract

The genome sequences of *Methylomonas methanica* (NCIMB 11130^T^, R-45363, and R-45371), *Methylomonas koyamae* (R-45378, R-45383, and R-49807), *Methylomonas lenta* (R-45370), and *Methylosinus* sp. (R-45379) were obtained. These aerobic methanotrophs were isolated from terrestrial ecosystems, and their distinct phenotypes related to nitrogen assimilation and dissimilation were previously reported.

## GENOME ANNOUNCEMENT

Microbial methane oxidation is the main methane sink in terrestrial environments. Aerobic methanotrophic bacteria can be physiologically diverse, especially in regard to the assimilation and dissimilation of various nitrogen compounds (1–3). Here, we report eight genome sequences of methanotrophs isolated from different terrestrial environments and that were previously found to represent different phenotypes based on their nitrogen metabolism (4). The type strain of *Methylomonas methanica* was acquired from NCIMB and was originally isolated from freshwater sediment (5, 6). Other *M. methanica* strains (R-45363 and R-45371) and *Methylomonas lenta* R-45370 were isolated from the top layer of a denitrification tank of a Belgian wastewater treatment plant (7, 8). *Methylomonas koyamae* R-45378 and R-45383 and *Methylosinus* sp. strain R-45379 were isolated from a wetland near Ghent, Belgium (8). *M. koyamae* R-49807 was isolated from a facultative waste stabilization pond in South Africa.

Genomic DNA from the eight strains was prepared using the guanidium-thiocyanate-EDTA-Sarkosyl method (9). DNA sequence data were obtained at BaseClear B.V., The Netherlands, using the Illumina HiSeq platform. The draft genomes were assembled using CLC Genomics Workbench 6.5. Details on the genomes are given in Table 1.

**TABLE 1:** General genome statistics and accession numbers

Genus/species	Strain^a^	No. of contigs	Genome size (Mb)	Genome coverage (×)	Mean G+C content (%)	Core nitrogen metabolism^b^	Previously assigned nitrogen phenotype (Hoefman)	NCBI accession no.
*M. methanica*	NCIMB 11130^T^	115	5.02	156	50.7	Nif, Nas, Gln, Nar, Nap, NirS, NirK, cNor	I	LUUF00000000
	R-45363 (LMG 26612)	137	5.41	90	51.3	Nif, Nas, Gln, NirS, cNor	III	LUUG00000000
	R-45371 (LMG 26614)	120	5.48	89	51.3	Nif, Nas, Gln, Nar, NirS, NirK, cNor	IV	LUUH00000000
*M. lenta*	R-45370 (LMG 26613)	168	4.7	83	46.6	Nif, Nas, Gln, NirS, cNor	VII	LUUI00000000
*M. koyamae*	R-45378 (LMG 26261)	137	5.11	68	56.1	Nif, Nas, Gln, NirS, cNor,	II	LUUJ00000000
	R-45383 (LMG 26263)	234	5.41	97	55.8	Nif, Nas, Gln, NirS	V	LUUK00000000
	R-49807 (LMG 27769)	144	5.18	90	55.9	Nas, Gln, NirS, cNor, qNor	VI	LUUL00000000
*Methylosinus* sp.	R-45379 (LMG 26262)	319	4.97	107	64.4	Nif, Nas, Gln	VIII	LUUM00000000

a Strains are publicly available in the BCCM/LMG bacterial collection. Strain collection numbers are given in parentheses.

b Nif, Nitrogen fixation; Nas, nitrate assimilation; Gln, ammonium assimilation; Nar, membrane-bound dissimilatory nitrate reduction; Nap, dissimilatory nitrate reduction; NirS, cytochrome *cd*_1_-dependent nitrite reductase; NirK, copper-dependent nitrite reductase; Nor, cytochrome *c*-dependent nitric oxide reductase; qNor, quinol-dependent nitric oxide reductase.

All eight sequenced terrestrial methanotrophs are obligate methane and methanol utilizers. All the genomes harbor genes typical for methanotrophs, including genes encoding particulate methane monooxygenase (*pmoCAB*) and the PQQ-dependent methanol dehydrogenases (*mxaFI*). The genomes of strains R-45363, R-45371, R-45370, R-45383, and R-45379 also contain the genes for the soluble methane monooxygenase (*mmoXYBZDC*). As expected, the gene inventory for nitrogen metabolism was strain dependent (Table 1). Genes involved in ammonium and nitrate assimilation are present in all genomes. All genomes except that of *M. koyamae* R-49807 also contain the complete operon for nitrogen fixation. Despite being obligate aerobic bacteria, all *Methylomonas* strains have the genomic potential for dissimilatory nitrate and/or nitrite reduction, as previously reported for *Methylomonas denitrificans* (10). *M. methanica* NCIMB 11130^T^ displays the most genetic redundancy to convert nitrate to nitrous oxide, with genes for both the membrane-bound cytoplasmic (*narGHJI*) and the periplasmic nitrate reductase (*napCBADF*), both the copper-dependent (*nirK*) and the cytochrome *cd*_1_-dependent nitrite reductase (*nirS*), and the cytochrome-dependent nitric oxide reductase. The other strains have various combinations of these nitrogen dissimilation genes, with *M. koyamae* R-49807 also harboring the gene for quinol-dependent nitric oxide reductase, while R-45383 does not encode any nitric oxide reductase. *Methylosinus* sp. R-45379 seems limited to the assimilation of various nitrogen compounds.

These genomes provide a valuable resource to link previously observed phenotypes to genomic inventory, gather new insights into the redundancy and environmental controls of nitrogen metabolism in closely related methanotrophs and the interplay between nitrogen and one-carbon metabolism.

### Nucleotide sequence accession numbers.

The genome sequences have been deposited in GenBank under accession numbers listed in Table 1.
